# Antioxidant Effect of Fructus Ligustri Lucidi Aqueous Extract in Ovariectomized Rats Is Mediated through Nox4-ROS-NF-κB Pathway

**DOI:** 10.3389/fphar.2017.00266

**Published:** 2017-05-22

**Authors:** Lili Wang, Rufeng Ma, Yubo Guo, Jing Sun, Haixia Liu, Ruyuan Zhu, Chenyue Liu, Jun Li, Lin Li, Beibei Chen, Liping Sun, Jinfa Tang, Dandan Zhao, Fangfang Mo, Jianzhao Niu, Guangjian Jiang, Min Fu, Dieter Brömme, Dongwei Zhang, Sihua Gao

**Affiliations:** ^1^Cell and Biochemistry Lab, Preclinical Medicine School, Beijing University of Chinese MedicineBeijing, China; ^2^Chinese Material Medica School, Beijing University of Chinese MedicineBeijing, China; ^3^Modern Research Center for TCM, Beijing University of Chinese MedicineBeijing, China; ^4^The First Affiliated Hospital of He'nan TCM University, ZhengzhouHenan, China; ^5^Diabetes Research Center, Beijing University of Chinese MedicineBeijing, China; ^6^The Research Institute of McGill University Health CenterMontreal, QC, Canada; ^7^Oral Biological Medicinal Science, University of British ColumbiaVancouver, BC, Canada

**Keywords:** *Fructus Ligustri Lucidi*, ovariectomy, NADPH oxidase 4 (Nox4), nuclear factor kappa B (NF-κB), oxidative stress

## Abstract

**Purpose:** This study is designed to explore whether *Fructus ligustri lucidi* (FLL) exhibits antioxidant effect in ovariectomized (OVX) rats, and to identify the signaling pathway involved in this process.

**Methods:** OVX rats were treated with FLL aqueous extract (3.5 g/kg) for 12 weeks. Serum, uteri, and tibias were harvested from the rats and the levels of total antioxidant capacity (TAC), nitric oxide (NO), malondialdehyde (MDA), 8-hydroxy-desoxyguanosine (8-OHdG), and superoxide dismutase (SOD) were determined. Changes in the levels of NF-κB-p65, phosphorylation of NF-κB-p65 (NF-κB-pp65), NF-κB inhibitor alpha (IκBα), phosphorylation of IκBα (p-IκBα), and NADPH oxidase 4 (Nox4) in uteri and tibias were determined by western blot, immunofluorescent and immunohistochemical analysis, respectively. In addition, the expression of cytochrome C (Cyto-C) and B-cell lymphoma-2 (Bcl-2) were determined in the tibias of rats. Histopathological changes in the bones were evaluated by hematoxylin-eosin staining. Bone mineral density (BMD) was determined in rat femurs by dual X-ray absorptiometry.

**Results:** Treatment of OVX rats with FLL aqueous extract improved redox homeostasis by increasing the levels of TAC and NO as well as decreasing the levels of MDA and 8-OHdG in serum, tibias, and uteri. Further, FLL extract also downregulated the expression of Nox4, NF-κB-p65, NF-κB-pp65, and p-IκBα in the uteri and tibias. Furthermore, administration of FLL–OVX rats increased Bcl-2 expression and prevented cytoplasmic release of mitochondrial Cyto-C in the tibias. In addition, FLL treatment also improved bone microstructure and increased cortical bone thickness as well as increased BMD values in the femurs of OVX rats.

**Conclusions:** FLL treatment may suppress oxidative stress response in OVX rats via regulating the Nox4/ROS/NF-κB signaling pathway. These results suggest the potential of using FLL as a natural antioxidant agent in preventing the development of osteoporosis.

## Introduction

Oxidative stress as a contributory factor of the imbalance between bone resorption and formation has received increasing attention for our understanding of post-menopausal osteoporosis (Cervellati et al., 2014; Li J. et al., 2015; Yazgan et al., 2016). Post-menopausal estrogen deficiency facilitates the redox imbalance and oxidative stress amplification (Lean et al., 2003) that stimulates osteoclastogenesis and inhibits osteoblastogenesis (Cornelius et al., 2014). Emerging evidence supports the concept that NADPH oxidase 4 (Nox4) is a constitutive enzyme specialized in controlling oxidative stress response (Cornelius et al., 2014), and that it serves as an oxygen sensor to generate reactive oxygen species (ROS) from molecular oxygen (Yong et al., 2013). Up-regulation of Nox4 may potentiate oxidative stress and induce mitochondrial dysfunction (Kuroda et al., 2010). Moreover, the elevated malondialdehyde (MDA) level and myeloperoxidase activity, and the reduced superoxide dismutase (SOD) level and catalase activity (Yalin et al., 2012) disrupt the redox status in the body, which will further trigger NF-κB activation and facilitate the release of cytochrome C (Cyto-C) from mitochondria into cytosol as well as osteoclastogenesis (Zhang R. et al., 2014; Strom et al., 2016).

Treatment with antioxidants will cool down the critical adaptation of oxidative stress, and subsequently contributes to the management of osteoporosis (Spilmont et al., 2014; Law et al., 2016). It has been revealed that antioxidant-based dietary approach is beneficial to osteoporosis prevention and treatment in Brazilian women (De Franca et al., 2013). In addition, attenuation of H_2_O_2_ stimulation increases osteoblasts viabilities and differentiation (Yan et al., 2017) through improving the mitochondrial redox homeostasis and functions (Dai et al., 2017). Elimination of ROS generation inhibits bone resorption through receptor activator of nuclear factor κB (NF-κB) ligand (RANKL) mediated NF-κB activation (Thummuri et al., 2015). Further, emerging evidence suggests that Chinese herbal medicines may exhibit anti-osteoporotic effects through an improvement of antioxidant activity (Rufus et al., 2013; Huang et al., 2015).

The fruits of *Ligustri Lucidi* (*Fructus Ligustri Lucidi*, FLL), known as NvZhenZi (Chinese Pinyin name) in Chinese, have been first recorded as a treatment option for osteoporosis in the book of *Sheng Nong's Herbal Classic* (Leung and Siu, 2013). We and others demonstrate that FLL improves Bone mineral density (BMD) and bone microstructure as well as bone mechanical strength in both aged (Ko et al., 2010) and growing female rats (Feng et al., 2014) as well as ovariectomized (OVX) rats (Zhang et al., 2006; Lyu et al., 2014; Guo et al., 2016a). FLL exhibited bone protective effects by improving calcium absorption and balance via increasing gene expression of transient receptor potential vanilloid 6 and calcium-binding protein-9k, and by decreasing renal calcium-sensing receptor gene expression (Zhang Y. et al., 2014; Zhang et al., 2015) via stimulating 1.25(OH)_2_D_3_/vitamin D receptor signaling (Feng et al., 2014) through inducing the activity of renal 25-hydroxyvitamin D-1α hydroxylase (Dong et al., 2010), as well as by stimulating parathyroid hormone production in mature normal female rats (Dong et al., 2012) and in type 1 diabetic mice (Zhang Y. et al., 2014). Meanwhile, FLL was also demonstrated to promote osteogenesis by stimulating the alkaline phosphatase (ALP) activity and accelerating the mineralization in human mesenchymal stem cells (Li et al., 2010) and UMR-106 cells (Wang et al., 2011). However, little is known about the effect of FLL on oxidative stress in OVX rats. In fact, Nox4 is widely expressed in bone (Fu et al., 2015) and uteri (Fletcher et al., 2014). Additionally, FLL has been demonstrated to exhibit antioxidant activity *in vitro* (Chen et al., 2013). Ovariectomy aggravates bone loss partly through disturbing redox homeostasis (Huang et al., 2014). In the light of these findings, we investigate whether FLL aqueous extract exhibits antioxidant effect in OVX rats and its potential association with the Nox4-ROS-NF-κB signaling pathway.

## Materials and methods

### Chemicals and antibodies

Total antioxidant capacity (TAC) kit (Cat. No: S0119), lipid peroxidation MDA assay kit (Cat. No: S0131), and total SOD assay kit (Cat. No: S0109) were purchased from Beyotime Institute of Biotechnology (Haimen, Jiangsu, China). Nitric oxide (NO) kit (Cat. No: A012) was obtained from Nanjing Jiancheng Bioengineering Institute (Nanjing, China). 8-hydroxy-2′-deoxyguanosine (8-OHdG) ELISA kit was obtained from Beijing Fangcheng Biotechnology Company (Beijing, China). Rabbit anti-Nox4 polyclonal antibody (Cat. No: NB110-58849) was obtained from Novus Biologicals (Littleton, CO, USA). Mouse anti-Cyto-C monoclonal antibody and mouse anti-p-IκBα monoclonal antibody (Cat. No: sc-8404) were from Santa Cruz Biotechnology (Dallas, TX, USA). Antibodies against NF-κB-p65 (ab16502), NF-κB-pp65 (ab86299), IκBα (ab32518) were obtained from Abcam Biocompany (Cambridge, MA, USA). Rabbit anti-Bcl-2 polyclonal antibody was obtained from Cell signaling (Danvers, MA, USA). Estradiol valerate (17-beta estradiol) tablets were purchased from Bayer Chemical Company (Leverkusen, Germany). All other reagents, except specially identified, were from Sigma (St. Louis, MO, USA).

### Preparation of FLL aqueous extract and characterization of single compounds

*Fructus ligustri lucidi* (FLL) was purchased from Beijing TongRenTang pharmacy (Beijing, China) and authenticated by Professor Zexin Ma [TCM museum at Beijing University of Chinese Medicine (BUCM)]. One hundred grams of raw FLL was grinded into powder and dissolved in 1000 ml of distilled water by continuous stirring for 48 h at 4°C. Then the aqueous extract was collected by centrifugation (4000 rpm at 4°C for 10 min). The supernatants were harvested and lyophilized to obtain a powder (20 g, 1 g contains 5 g raw FLL).

The sample was analyzed by an HPLC-DAD-ESI-MS^n^ (SHIMADZU, Japan), which was equipped with a DAD detector (SPD-M10AVP, SHIMADZU) and IT-TOF-MS. HPLC conditions: column, DIKMA (C18, 4.6 × 250 mm, 5 μm); column temperature, 25°C; mobile phase, methanol (A)-water (B) with gradient elution, 0–15 min, 5 → 25%A; 15–24 min, 25%A; 24–32 min, 25 → 50%A; 32–50 min, 50%A; 50–55 min, 50 → 95%A; flow rate, 0.8 ml/min. The injection volume was 20 μl. The wavelength was set at 220 nm. MS condition: positive ion mode; nebulizing gas, N_2_, flow rate, 1.5 ml/min; drying gas, N_2_, pressure, 100 MPa; detector voltage, 1.40 kV; CDL pressure, normal mode; CDL temperature, 200°C; block heater temperature, 200°C; interface voltage 1.4 kV; IT vacuum, 1.9 × 10^−2^ Pa; cooling gas and collision-induced dissociation (CID) collision gas, Ar; Automatic multilevel MS^1^, MS^2^, and MS^3^ full scan; ion accumulation time, 100 ms; CID collision energy, 50%.

### Animals

Forty 3-month-old female Sprague Dawley rats (body weight, 220 ± 10 g) were purchased from Beijing SiBeiFu Animal Technology Co. Ltd. (Beijing, China). The rats were housed in the clean level condition animal housing facilities (certification number SCXK (Jing) 2011-0024) of BUCM, temperature of 22 ± 1°C, humidity of 55 ± 5%, and a 12 h light/dark cycle with free access to tap water and chow. All the protocols were reviewed and approved by the Animal Care Committee of BUCM, China.

### Experimental design

After acclimation for 1 week, the OVX models were established by removing the bilateral ovaries from the (1% sodium pentobarbital, 0.4 ml/100 g, i.p.) rats. The sham operated control group (*n* = 9) rats were performed by removing same amount of fat tissues around the ovaries. One week after surgery, OVX rats were randomly divided into 3 groups of 9 rats each. All the experimental rats were grouped and treated for 12 weeks as followings:

**Group 1:** Sham operated control (Sham) group: The sham operated rats were daily administered with equal volume of distilled water by gavage.
**Group 2:** OVX control (OVX) group: The OVX rats were daily administered with equal volume of distilled water by gavage.
**Group 3:** OVX plus Estradiol valerate treated (EV)group. The OVX rats were daily administered with estradiol valerate (0.1 mg/kg) by gavage.
**Group 4:** OVX plus *Fructus ligustri lucidi* treated (FLL) group. The OVX rats were daily administered with FLL aqueous extract [3.5 g (raw FLL)/kg; Guo et al., 2016a] by gavage.

At the end of the 12-week treatment, the rats were fasted for 12 h, and then euthanatized with 1% sodium pentobarbital (0.4 ml/100 g, i.p.). Subsequently, blood was collected from the heart by puncture, and the uteri, tibias and femurs were harvested from the rats. Blood serum was prepared by centrifugation. All the specimen samples for biochemical and histological analysis were either stored at −80°C or prepared for tissue sectioning.

### Oxidant and antioxidant parameters examination

Tissues were lyophilized and ground, and then homogenized in PBS buffer and centrifuged (1,000 × g, 10 min). Supernatants were used for all biochemical assays described herein. Biochemical results in the tissues were normalized to protein content using BCA protein assay kit (Applygene; Beijing, China).

The levels of TAC, NO, MDA, 8-OHdG, and SOD in serum and homogenates of the uteri and tibias were evaluated by the appropriate biochemical analysis kits according to the manufacture's protocols. TAC, MDA and NO levels were expressed in mmol/L or μmol/L of serum or per gram protein in tissues. 8-OHdG levels were evaluated by ELISA assay and the results were expressed in ng per liter of serum and ng per gram protein in tibias. SOD activity was expressed in units per mL (U/mL).

### Histopathological examination

Hematoxylin and eosin (H&E) staining was conducted according to the protocol as previously described (Guo et al., 2016b). After staining, the slides were used for evaluating bone microarchitecture changes, including the structure and morphology of trabecular bone and lipid droplets. Images were captured using Olympus BX53 fluorescence microscope (Tokyo, Japan) and histomorphometric measurements were carried out with a semi-automated image analysis system using CellSens (V1.5, Olympus) according to the methods recommended by the ASBMR Histomorphometry Nomenclature Committee (Dempster et al., 2013). The trabecular area below 1 mm cartilage was measured with a digital meter. Cortical bone thickness was examined in the mid-diaphyseal region of the femur at regular intervals of 80 μm with a digital micrometer. As for the measurements, at least 8 sections from different rats in each group were selected for quantification by two examiners.

### Immunohistochemical staining and confocal microscopy for NF-κB nuclear translocation

Immunohistochemical (IHC) staining was conducted according to the procedure as previously described (Guo et al., 2016b) with some modifications. Briefly, 5 μm longitudinal sections of the paraffin embedded uteri and tibias were kept in the oven at 60°C for 24 h and then followed by defatting with xylene and hydrating with graded ethanol (100–70%). Then slides were sequentially incubated with an antigen retrieval solution (Shanghai ShunBai Biotechnology Company, China), 3% H_2_O_2_ for 30 min, and incubated with a primary antibody [NF-κB (1:50) or Nox4 (1:100)] overnight at 4°C. For negative controls, the primary antibodies were replaced by non-immunized goat serum. On the next day, the slides for IHC staining were incubated with corresponding secondary antibodies (Beijing Biosynthesis Biotechnology Co. Ltd., China) for 30 min followed by DAB and hematoxylin staining. Meanwhile, the slides for immunofluorescence staining were incubated with fluorescein-conjugated goat anti-rabbit IgG and followed by DAPI staining. Finally, the slides of IHC staining were examined and photographed using Olympus BX53 fluorescence microscope. The intensity of DAB staining was analyzed using Image Pro Plus 6.0 software. The immunofluorescence staining was imaged using a NIKON UltraVIEW VoX: 13UV003 confocal system (Nikon; Tokyo, Japan).

### Mitochondria isolation from bone

For mitochondria isolation, the tibia was pulverized in liquid nitrogen and then fractionated using a tissue mitochondria fractionation kit (Beyotime Biotechnology; Jiangsu, China) according to manufacturer's instructions. Briefly, the powder of tibia was treated with 10 volumes of PBS, followed by centrifuging at 600 g for 20–30 s at 4°C. Then the pooled pellets were incubated with pre-cooled trypsin solution for 20 min followed by centrifuging at 600 g for 20–30 s at 4°C. After that, the pellets were further incubated with 8 volumes of mitochondria separating solution and homogenized for 20–30 times followed by spinning at 1,000 g for 10 min at 4°C. Then, the harvested supernatant were further centrifuged at 11,000 g for 10 min at 4°C. Subsequently, the harvested supernatants was further centrifuged at 12,000 g for 10 min at 4°C. The pellets were considered as the mitochondrial fraction. The resultant supernatants devoid of mitochondria were pooled for Cyto-C assay (western blot assay).

### Western blot assay

The homogenates of uteri and tibias were prepared by lyophilizing and grinding followed by lysing in a buffer containing 20 mM Tris–HCl, pH 7.5, 0.1% (v/v) Igepal, 6 mM sodium deoxycholate, 150 mM NaCl, 2 mM ethyleneglycoltetraacetic acid (EGTA), 2 mM EDTA, 0.1 mM Na_2_SO_4_, 20 mM NaF, and a protease inhibitor cocktail tablet (Roche, German) for 30 min at 4°C. Then, the supernatants and pellets in the homogenates were separated by spinning at 14,000 g for 20 min at 4°C and protein concentrations were determined using BCA protein assay kit. Next, 100 μg of protein from the uteri and tibias homogenates were separated on 12 or 15% discontinuous SDS-PAGE gel followed by transferring onto nitrocellulose membrane. Then the membrane was subsequently incubated with appropriate primary antibodies [Nox4 (1:500), NF-κB-p65 (1:1,000), NF-κB-pp65 (1:1,000), IκBα (1:1,000), p-IκBα and Cyto-C (1:500), respectively] overnight at 4°C, and the corresponding HRP labeled secondary antibodies for 1 h at room temperature. Immunopositive bands were visualized with high sensitivity ECL luminous liquid and the images were captured with Azure Bio-imaging systems (California, USA). The gray values of the bands were quantified using the Image J software, and normalized with the corresponding β-actin (1:5,000) as the internal control.

### BMD determination

Bone mineral density (BMD) values at the metaphysis of the rat femurs were determined *ex vivo* with a dual energy X-ray absorptiometry (DEXA, Discovery Wi; Hologic, Bedford, MA, USA), which was equipped with a small animal protocol software program.

### Statistical analysis

The results were expressed as mean ± SD. One-way ANOVA test was performed between multiple groups when homogeneity of variance and normality were met using SPSS software (Version 20.0). Otherwise, Dunnett's T3 and Nonparametric tests were conducted between multiple groups, respectively. A value of *p* <0.05 was considered to be statistical difference.

## Results

### FLL improved redox homeostasis in serum and homogenates of the tibias and uteri of OVX rats

**Figure 1:**
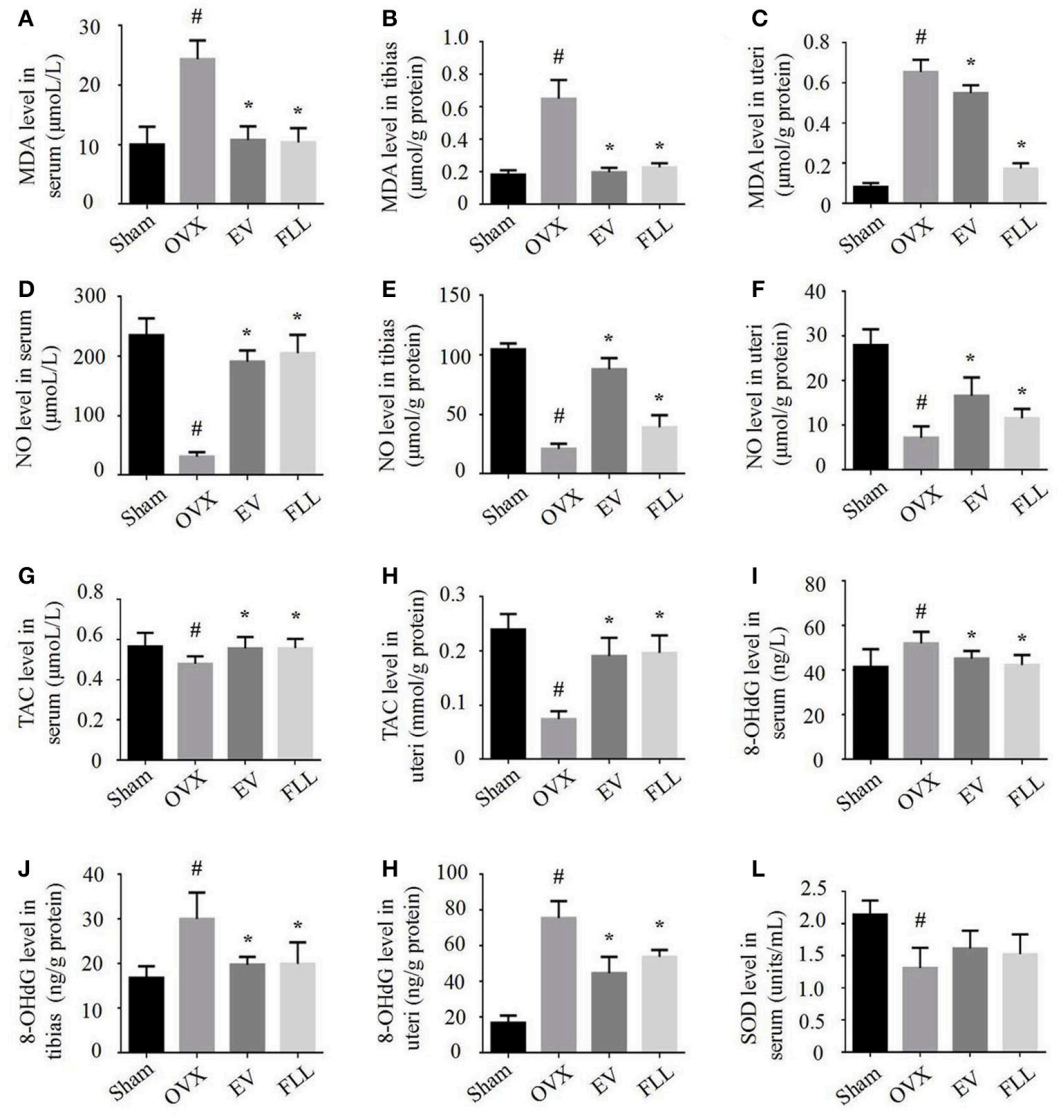
**FLL treatment improved the levels of MDA (A–C)**, NO **(D–F)**, TAC **(G,H)**, 8-OHdG **(I–K)**, and SOD **(L)** in serum, uteri and tibias of OVX rats. 9 samples per group were taken for each assay. Data are presented as mean ± SD. ^#^*p* <0.05 with Sham group, *p <0.05 compared with OVX group.

The delicate interplay between oxidant and antioxidant systems plays a critical role in maintaining body health and function (Rahal et al., 2014). As shown in Figures 1A–C, relative to vehicle treated OVX rats, EV, and FLL treatment of OVX rats attenuated the increase in MDA level in serum by 55.6 and 57.3%, in tibias by 68.3 and 63.5%, and in uteri by 15.4 and 73.8%, respectively. Meanwhile, administration of FLL to OVX rats also significantly increased NO level in serum (Figure 1D), tibias (Figure 1E), and uteri (Figure 1F) in comparison with vehicle treated OVX rats (*p* <0.05). Additionally, TAC levels were also significantly increased in the serum (Figure 1G) and uteri (Figure 1H) of FLL and EV group rats (*p* <0.05), as compared to that in vehicle treated OVX rats.

8-OHdG has been widely accepted as a biomarker for free radical-induced oxidative DNA stress (Valavanidis et al., 2009). In our experiment, we found that EV and FLL treatments attenuated the elevations of 8-OHdG in serum (Figure 1I), tibias (Figure 1J), and uteri (Figure 1K) in rats by 13.0 and 18.5%, 34.1 and 33.4%, 40.8 and 28.8%, respectively, when compared to vehicle treated OVX rats. However, FLL treatment did not significantly elevate SOD activity in serum (Figure 1L) of OVX rats compared with vehicle treated OVX rats. The results suggest that both FLL and EV exhibit antioxidant activity in OVX rats. FLL seems to be more potent in reducing the MDA level in OVX rats than EV.

### Effect of FLL on NF-κB expression in uteri and tibias of OVX rats

**Figure 2:**
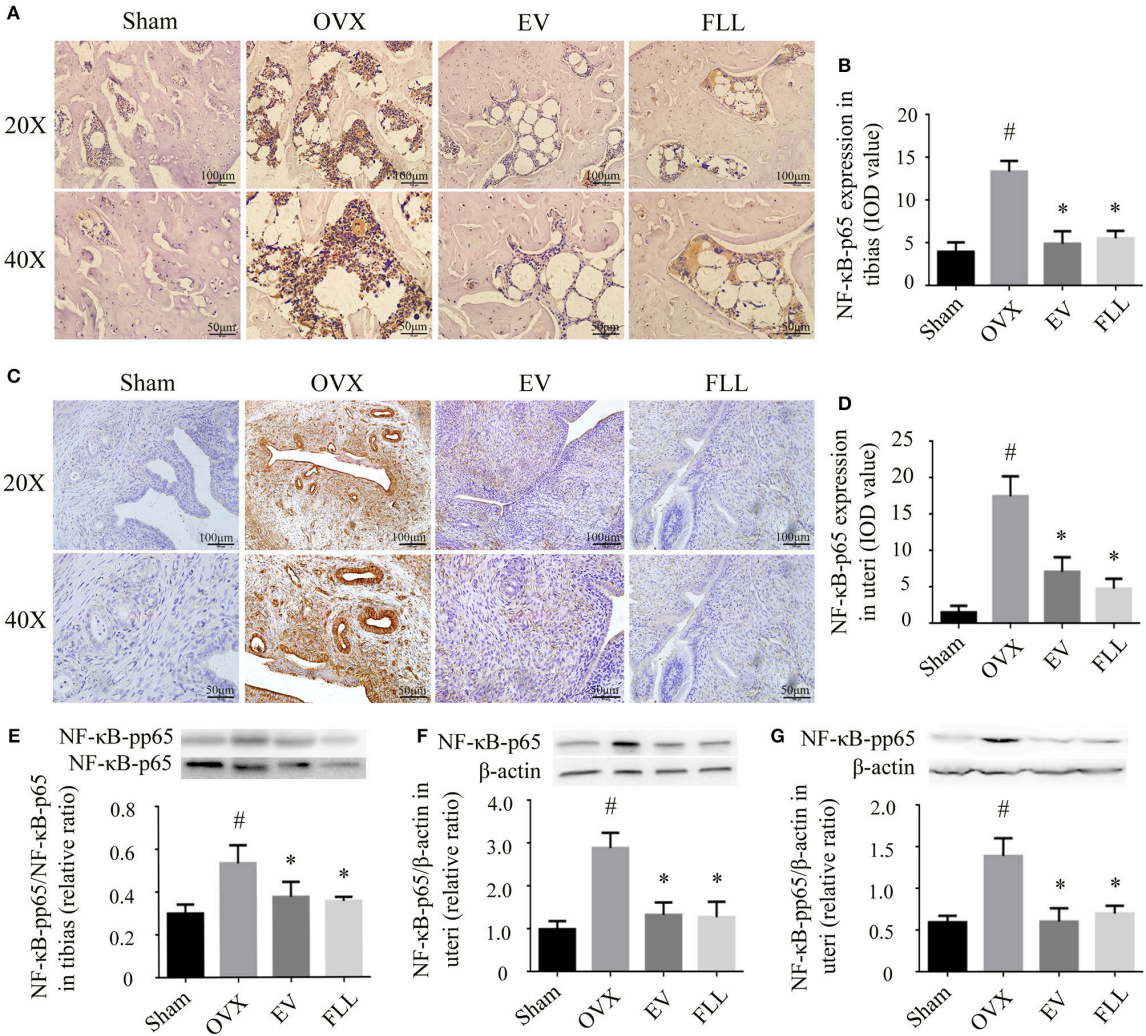
**The representative immunohistochemical staining** (**A–D**; sections were counterstained with hematoxylin; original magnification, × 20) and western blot analysis **(E–G)** showed that FLL treatment decreased NF-κB-p65 and NF-κB-pp65 expression in tibias and uteri of OVX rats (*n* = 9). Data are presented as mean ± SD. IOD denotes integrated optical density of interested areas. ^#^*p* < 0.05 with Sham group, *p < 0.05 compared with OVX group.

Exposure to oxidative stress may cause activation of the transcription factor NF-κB, which further contributes to osteoporosis (Callaway and Jiang, 2015). Therefore, we then determine whether the FLL aqueous extract affects NF-κB activation. As shown in Figures 2A–D, IHC staining revealed that NF-κB-p65 expression was significantly increased in the tibias as well as in the uteri of OVX rats relative to that of sham control group (*p* <0.05). After the rats were treated with FLL or EV for 12 weeks, both the intensity and extent of staining for NF-κB-p65 were significantly reduced in the tibias and uteri, suggesting that FLL treatment significantly decreased NF-κB-p65 expression in these tissues when compared to vehicle treated OVX rats (*p* < 0.05).

Phosphorylation of NF-κB-p65 also contributes to the activation of NF-κB that further triggers the oxidative insults (Shie et al., 2015). As shown in Figure 2E, either FLL or EV treated OVX rats for 12 weeks showed a markedly decreased ratio of NF-κB-pp65/NF-κB-p65 in the tibias as compared to vehicle treated OVX rats (*p* < 0.05). A similar trend toward the levels of NF-κB-p65 and NF-κB-pp65 was also observed in the uteri of OVX rats following FLL treatment (Figures 2F,G). Overall, FLL was comparable to that of EV in suppressing NF-κB activation in OVX rats. The results indicate that treatment with FLL significantly inhibits ROS-induced NF-κB-p65 activation and phosphorylation in OVX rats.

### Effect of FLL on IκBα expression in tibias and uteri of OVX rats

**Figure 3:**
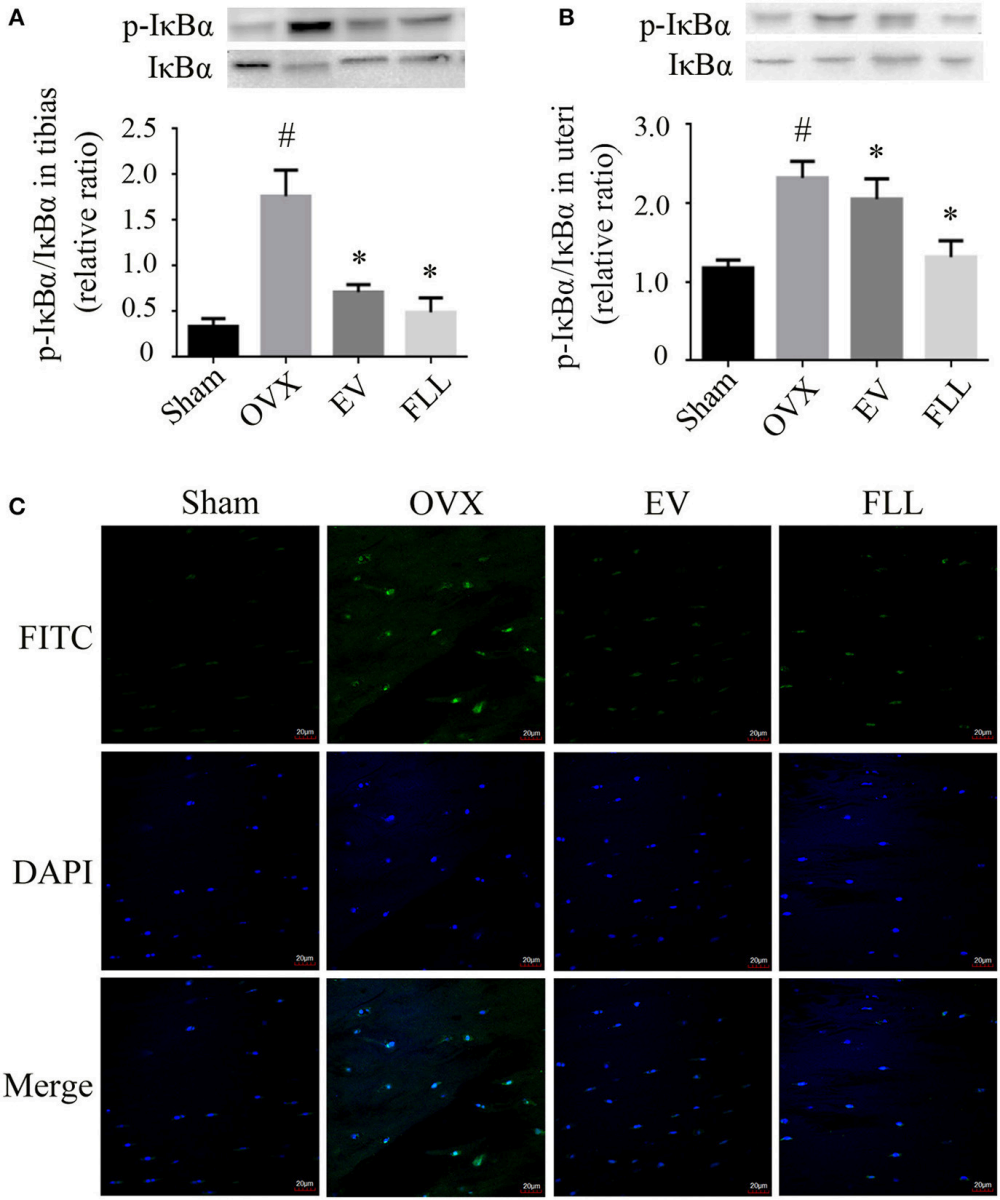
**The representative western blot images and their analysis showed that FLL treatment decreased IκBα and p-IκBα expression in the tibias (A)** and uteri **(B)** of OVX rats (*n* = 9). In addition, confocal microcopy of immunofluorescence staining (**C**; original magnification, × 60) showed that FLL blocked NF-κB-p65 nuclear translocation in the femurs of OVX rats. The green color represents NF-κB-p65 staining, the blue color represents nuclei staining, and the cyan (greenish-blue) color represents nuclear translocation. ^#^*p* < 0.05 with Sham group, *p < 0.05 compared with OVX group.

Phosphorylation of IκB is essential for NF-κB activation (Viatour et al., 2005) by regulating the NF-κB:IκB complex formation that is mainly found in the cytoplasm. Subsequently, we investigated the changes of IκBα expression in the cytoplasm of the tibias and uteri in OVX rats. As shown in Figures 3A,B, administration of FLL and EV to OVX rats for 12 weeks significantly reduced p-IκBα expression in the tibias and uteri as compared to vehicle treated OVX rats (*p* < 0.05). FLL is more efficient in reducing p-IκBα/IκBα ratio in OVX rats than EV. The results indicate that FLL inhibits the phosphorylation of IκBα and subsequently stabilizes the NF-κB:IκB complex in OVX rats.

### Effect of FLL on NF-κB nuclear translocation in the femurs of OVX rats

High level of oxidative stress activates NF-κB and then promotes its nuclear translocation, which may further contribute to osteoclastogenesis (Johnson et al., 2016). As shown in Figure 3C, NF-κB-p65 was retained in the cytoplasm in rat femurs of sham operated control group evaluated by immunofluorescence staining under confocal microscope. Upon OVX in rats, NF-κB-p65 was liberated from NF-κB:IκB complex to enter the nuclei, as evidenced by an increase in the intensity of greenish-blue color staining in the nuclei of rat femurs, suggesting that OVX induces NF-κB nuclear translocation in rats. Interestingly, FLL or EV treatment substantially inhibited NF-κB-p65 nuclear translocation, as evidenced by a decrease in the intensity of greenish-blue color staining.

### Effect of FLL on the Nox4 expression in tibias and uteri of OVX rats

**Figure 4:**
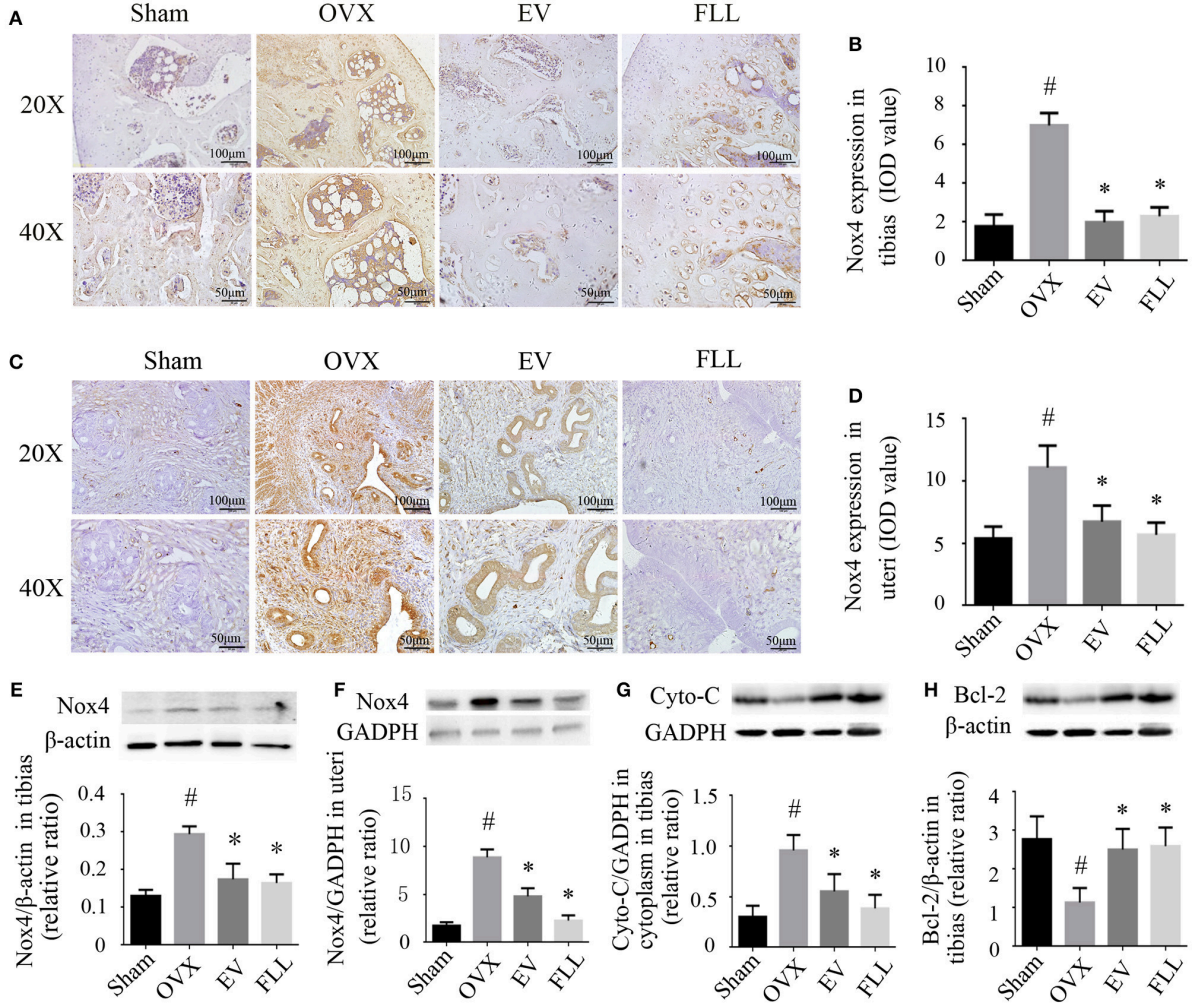
**The representative images of immunohistochemical staining (A–D;** sections were counterstained with hematoxylin; original magnification, × 20), and western blot assays **(E,F)** showed that FLL treatment decreased Nox4 expression in tibias and uteri of OVX rats (*n* = 9). In addition, FLL treatment also decreased cytochrome C (Cyto-C; **G**) and increased Bcl-2 expression **(H)** in the tibias of OVX rats (*n* = 9). Data are presented as mean ± SD. IOD denotes integrated optical density of interested areas. ^#^*p* < 0.05 with Sham group, *p < 0.05 compared with OVX group.

Nox4 is mainly expressed in osteoclasts and osteocytes of the bone (Goettsch et al., 2013; Hoff and Buttgereit, 2014), and in myometrial cells of the uteri (Fletcher et al., 2014), which is committed to catalyzing the reduction of molecular oxygen to various ROS. Therefore, we examine whether Nox4 expression is affected by FLL. As shown by western blot and immunohistochemistry analysis (Figures 4A–F), Nox4 expression was increased in the tibias and uteri of OVX rats in comparison to that of the sham operated control group (*p* < 0.05). After 12 weeks of treatment, Nox4 levels in the tibias and uteri of the FLL- and EV-treated group were markedly reduced as compared to that of rats in vehicle treated OVX group (*p* < 0.05). In addition, FLL has much greater effect on blocking Nox4 expression in the uteri of OVX rats than EV. The results suggest that FLL possesses the ability of scavenging ROS production through regulating Nox4 expression in OVX rats.

### Effect of FLL on the expression of Cyto-C and Bcl-2 in tibias of OVX rats

The increased production of ROS contributes to the release of Cyto-C from mitochondria into the cytosol (Liang et al., 2012), which facilitates cell apoptosis and osteoporosis (Xiong et al., 2015). Activation of NF-κB contributes to mitochondrial dysfunction, which further potentiates NF-κB activation (Cherry and Piantadosi, 2015). Western blot analysis (Figure 4G) revealed that ovariectomy led to higher expression of Cyto-C in the cytoplasm in the tibias of the vehicle treated OVX group rats when compared to that of the sham operated control group (*p* < 0.05). Treatment with FLL and EV for 12 weeks significantly prevented cytoplasmic Cyto-C release (*p* < 0.05) relative to vehicle treated OVX rats. It appears that FLL is much stronger in blocking Cyto-C release in the cytoplasm of tibias than EV.

Bcl-2 prevents mitochondrial membrane disruption, and inhibits Cyto-C activation and that of other pro-apoptotic factors (Czabotar and Lessene, 2010). As shown in Figure 4H, ovariectomy markedly reduced the expression of Bcl-2 in the tibias of the vehicle treated OVX group as compared to that of the sham operated control group (*p* < 0.05). Treatment with FLL or EV for 12 weeks significantly reversed this consequence in the tibias of OVX rats (*p* < 0.05). The results suggest that FLL could stabilize the mitochondria by recalling Bcl-2 and Cyto-C expression to near normal.

### Effects of FLL on bone pathological changes and BMD in OVX rats

**Figure 5:**
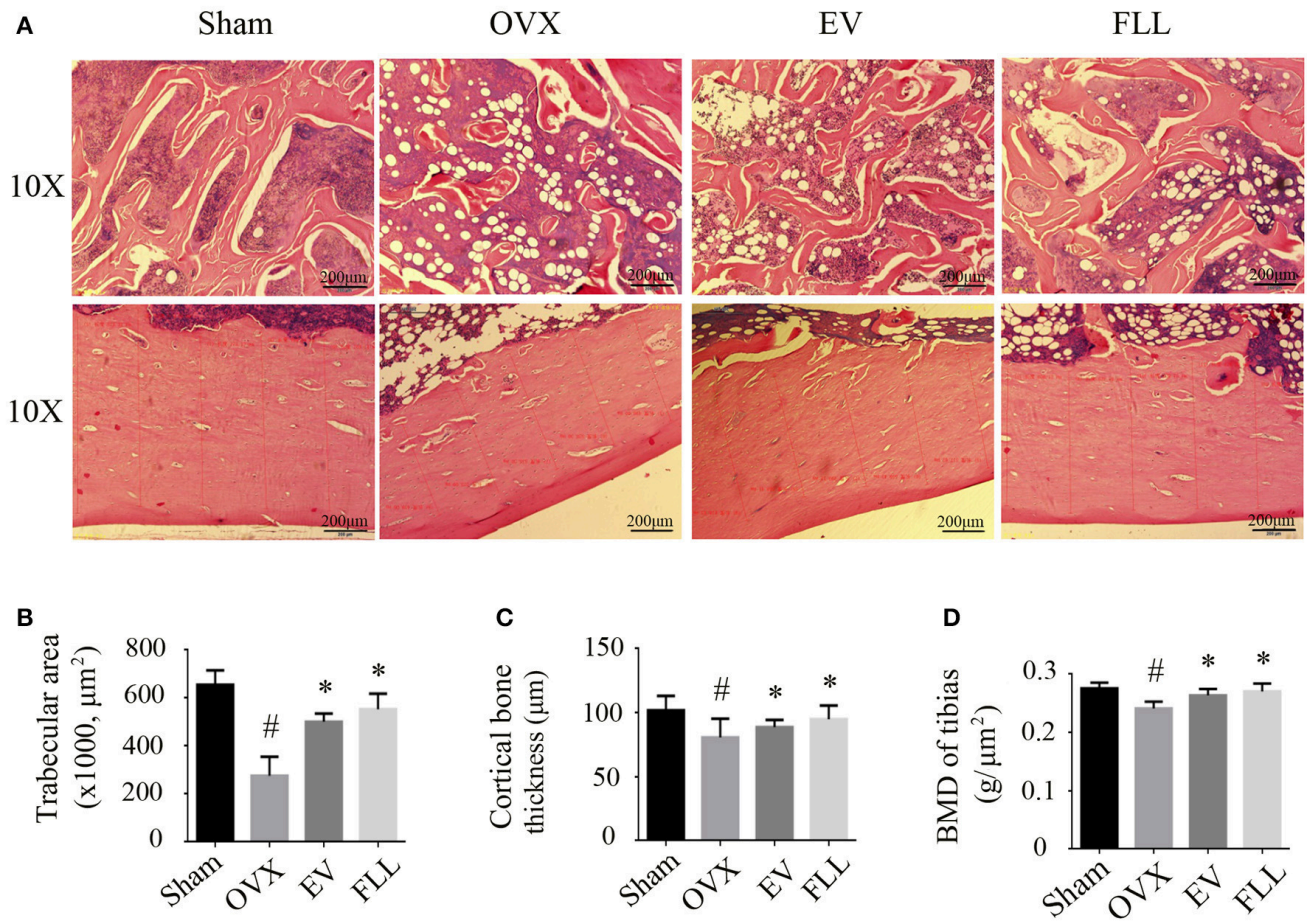
**The representative images of H&E staining and BMD measurements showed that FLL treatment significantly improved bone microstructures (A)**, increased trabecular bone area **(B)** and cortical bone thickness **(C)**, as well as improved bone mineral density (BMD) **(D)** in the femurs of OVX rats (*n* = 7). Data are presented as mean ± SD. ^#^*p* < 0.05 with Sham group, *p < 0.05 compared with OVX group.

Resistance to fracture depends not only on the size and distribution of the trabeculae, but also on the cortical bone thickness (Iolascon et al., 2014). As shown in Figure 5A, H&E staining showed that trabecular bone was formed as a dense and regular meshwork in the femurs of sham operated control group rats. After ovariectomy, trabecular bone lost its normal architecture and became thinner and discontinuous. In addition, trabecular bone area and cortical bone thickness were reduced, and the marrow cavity was increased in vehicle treated OVX rats (Figures 5B,C). Supplementation with FLL and EV to OVX rats for 12 weeks resulted in an improvement of trabecular bone area as seen in an increased regularity and thickness of trabecular bone as well as a decrease in marrow cavities. The cortical bone thickness was also significantly increased in the FLL treatment group relative to vehicle treated OVX rats (*p* < 0.05). Interestingly, the improvement of FLL on bone microstructures was more evident in FLL group rats than in EV group rats.

The bone preserving effect of FLL was also reflected in the BMD improvement in OVX rats. As illustrated in Figure 5D, ovariectomy produced the expected significant reduction in BMD when compared with that of sham operated control group (*p* < 0.05). Administration with FLL and EV to OVX rats for 12 weeks markedly increased BMD values, as compared to vehicle treated OVX rats (*p* < 0.05). These results suggest that FLL treatment clearly improves bone quality during the course of osteoporosis.

### Characterization of FLL aqueous extract

**Figure 6:**
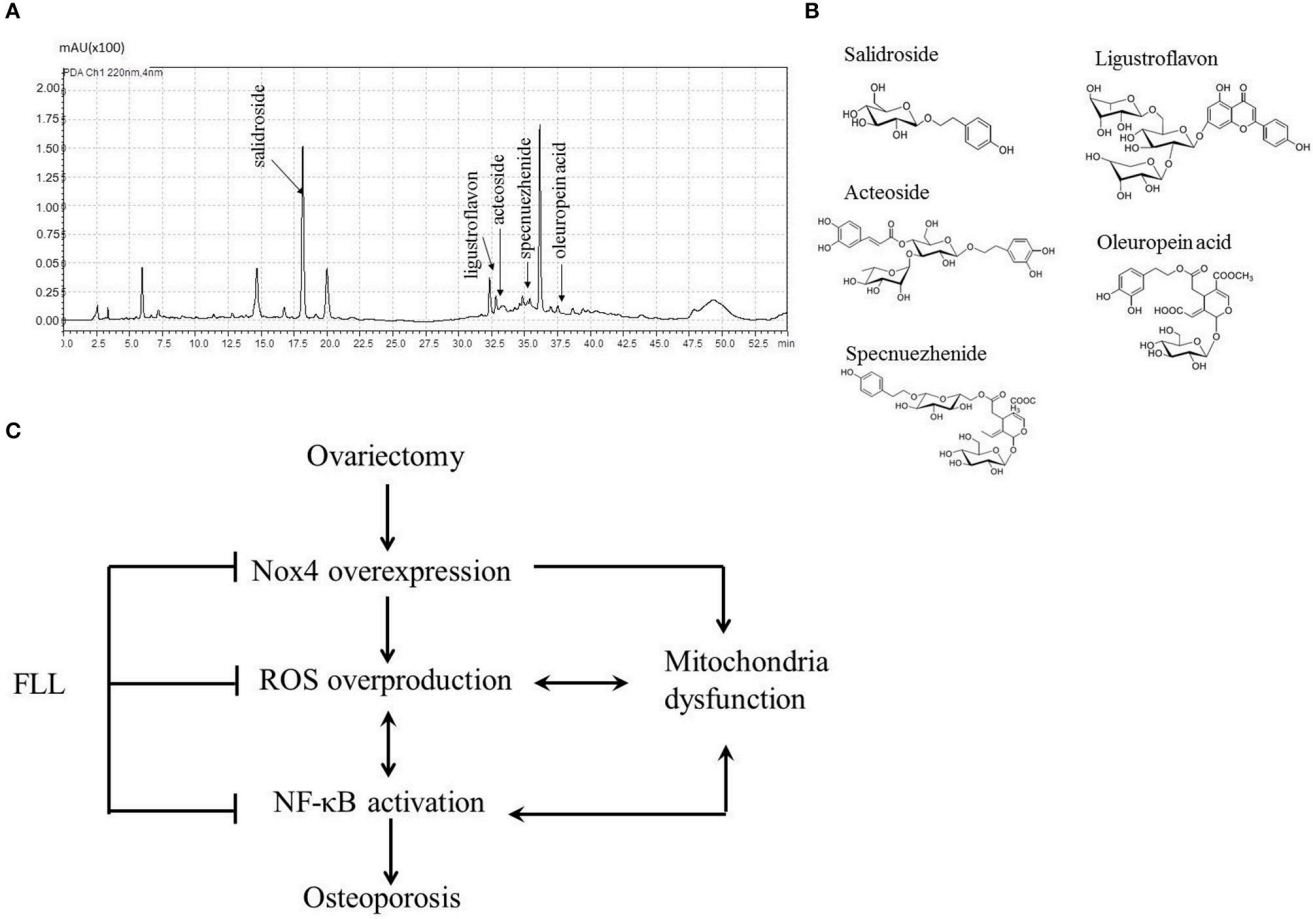
**Chromatograms profile (A)** and chemical structures **(B)** showed five single compounds from FLL aqueous extract using HPLC analysis at 220 nm. Schematic diagram **(C)** illustrated the underlying mechanisms of FLL against osteoporosis in OVX rats. Ovariectomy triggers Nox4 overexpression followed by ROS overproduction and NF-κB activation as well as mitochondria dysfunction, which initiates and accelerates osteoporosis. Administration of FLL to OVX rats prevents the development of osteoporosis through regulating Nox4 expression, scavengering abundant ROS and normalizing NF-κB expression as well as resuming mitochondria function.

As salidroside is one of the reference constituents of FLL aqueous extract, and has also been demonstrated to exhibit an antioxidant effect in SH-SY5Y osteoblast cells (Ju et al., 2012), we analyzed the ingredients in the FLL extract by HPLC-DAD–ESI-MS. As shown in Figures 6A,B, five constituents were identified, including salidroside, ligustroflavon, acteoside, specnuezhenide, and oleuropein acid.

## Discussion

The present study reveals that administration with FLL to OVX rats decreases MDA and 8-OHdG levels, increases SOD, NO, and TAC levels in serum, tibias and/or uteri, as well as reduces the expression of Nox4, NF-κB and p-IκBα, and regulates the expression of Cyto-C and Bcl-2 in the tibias and femurs, which results in an improvement of bone microstructure and mineral density.

Our observation from the current study and data from other experiments (Fu et al., 2015) demonstrate that ovariectomy results in an elevation of Nox4 gene expression and protein synthesis as well as an increase in ROS production. Considering that FLL could scavenge free radical oxygen (Jeong et al., 2016) and elevate antioxidant enzyme levels in human neuroblastoma SH-SY5Y cells (Ju et al., 2012), we then investigated whether FLL exhibited antioxidant activities in osteoporotic rats. As we expected, the results demonstrated that FLL treatment mitigated Nox4 overexpression and inhibited ROS production in OVX rats. In the previous study, we demonstrated that treatment of OVX rats with FLL markedly improved bone mechanical strength and bone microarchitectures (Guo et al., 2016a). Moreover, inhibition of Nox4 activity and expression further contributed to protection of osteoblasts against H_2_O_2_ insults and prevention of OVX-induced bone loss (Fu et al., 2015). Therefore, the findings of the current study may suggest that FLL exhibits bone protective effect through inhibiting ROS release via the regulation of Nox4 expression in OVX rats.

In the current study, we found that ovariectomy resulted in a dramatic increase in ROS production, which further led to an increase in p-IκBα and NF-κB-p65 expression. This is in line with Rajakumar's observation that oxidative stress triggers NF-κB-p65 activation by increasing the phosphorylation of IκBα and the degradation of cytoplasmic IκBα (Rajakumar et al., 2014). Further, inhibition of NF-κB activation suppresses bone resorption and further contributes to maintaining bone homeostasis in OVX mice (Chang et al., 2009). The results from the present study also demonstrate that FLL prevents bone loss by reducing NF-κB-p65 activation in OVX rats, suggesting that FLL treatment prevents oxidative stress *in vivo* through the inhibition of NF-κB activation.

Our findings also demonstrate that administration with FLL for 12 weeks to OVX rats decreases the expression of p-IκBα and NF-κB-p65 in the tibias and uteri. In addition, FLL treatment upregulates the Bcl-2 expression and inhibits the release of Cyto-C into the cytoplasm in the tibias of OVX rats. Similarly, FLL is demonstrated to possess the ability of inhibiting NF-κB activation and decreasing IκBα phosphorylation in mouse peritoneal macrophages (An et al., 2007). Further, the resultant estrogen deficiency by ovariectomy stimulates mitochondrial malfunctions for increasing ROS production (Yazgan et al., 2016) and Cyto-C expression (Li et al., 2014) as well as decreasing Bcl-2 expression (Lin et al., 2016). Moreover, mitochondrial dysfunction further facilitates NF-κB activation (Cherry and Piantadosi, 2015). Additionally, Bcl-2 affects the mitochondrial function by regulating Cyto-C release and blocking NF-κB activation (Czabotar and Lessene, 2010). Therefore, the present results may also indicate that FLL treatment regains the mitochondria function by inhibiting Cyto-C activation via resuming Bcl-2 expression, and then preventing NF-κB-p65 activation and phosphorylation via inhibiting Nox4 overexpression in OVX rats.

Ovariectomy triggers the pronounced myometrial and endometrial atrophy in the uteri, which may lead to an increase in Nox4 expression, and subsequent ROS generation and deterioration of redox homeostasis (Behr et al., 2012; Fu et al., 2015). 17β-estradiol has been demonstrated to reverse OVX induced oxidative stress in rats (Lee et al., 2005). In the current observation, we also show that supplement of OVX rats with EV or FLL for 12 weeks significantly reduces Nox4 expression, ROS generation and NF-κB activation in the uteri. FLL is more potent in eliminating oxidative stress than EV. Collectively, the present results suggest that FLL aqueous extract comprehensively exhibits antioxidant effect in the uteri and tibias in OVX rats, which contributes to improving uteri and bone health.

The current findings and the results from other groups (Zhang et al., 2008; Khosla, 2010) confirm that both FLL and EV could prevent bone loss through scavenging ROS and improving calcium balance in OVX rats. In addition, Zhang et al. (Chen et al., 2013) demonstrated that aqueous extract of FLL and its 20% ethanol fraction exhibited estrogenic activity via ERα and ERβ mediated estrogen response element gene expression in Hela cells. Further, Wang et al. (2011) found that the addition of ER antagonist (ICI182780) to aqueous extract of FLL treated UMR-106 osteoblasts effectively inhibited ALP activity. Moreover, the reported estrogenic activity may be associated with the trace amount of isoflavones (apigenin, luteolin, quercetin, etc) in FLL (Shen et al., 2007; Xu et al., 2007). In addition, FLL has the ability of promoting osteoblastogenesis and inhibiting osteoclastogenesis in cultures (Li Q. et al., 2015; Xu et al., 2016), similar to that of estrogen. However, estrogen therapy always accompanies with high risk of the side effects, such as breast cancer, uterine cancer, and stroke. In contrast, FLL does not show obvious side effects in the prevention of osteoporosis so far.

*Fructus ligustri lucidi* (FLL) contains a lot of active ingredients which exhibits antioxidant activities. Ju *et al*. demonstrated that the water extract of FLL showed free radical scavenging activity and an inhibition of lipid peroxidation (Ju et al., 2012) *in vitro*. Further, using ALP activity guided isolation assay, they found that tyrosol, tyrosyl acetate, hydroxytyrosol, salidroside, oleoside dimethyl ester, oleoside-7-ethyl-11-methyl ester, nuzhenide, and G13 exhibited anti-oxidative activities *in vitro* (Chen et al., 2013). In the current study, five compounds, including salidroside, ligustroflavon, acteoside, specnuezhenide, and oleuropein acid, were identified in the FLL aqueous extract. Altogether, these compounds may be responsible for the FLL antioxidant actions seen in OVX rats.

However, some limitations should be noted in the current study. Firstly, we did not observe the differences in the metabolites or enzymes between the rats in sham operated group and that in normal control group. However, Noorafshan et al. studied the biochemical and stereological parameters between the normal control and sham operated control groups (Noorafshan et al., 2015). They claimed that there were no statistical differences in serum calcium, phosphorus, and ALP levels as well as bone volume and total number of the osteocytes, osteoblasts, and osteoclasts between these two groups. Therefore, the rats in sham operated control group are always assumed similar as the rats in the normal control group during the evaluation of bone protective effects in most of experimental osteoporosis studies. Secondly, the TRAP staining was not performed in the bone section. TRAP staining is one of useful approaches to evaluate osteoclast activity. We have tried several commercial staining kits including sigma (387A-1KT) to staining to the slides, and the results were not successful. However, the previous study from our group demonstrated that FLL treatment reduced serum C-telopeptide of type I collagen (CTX-I) in OVX rats (Guo et al., 2016a). Increased CTX-I level is associated with osteoclasts activities and bone resorption (Henriksen et al., 2007). Future endeavors are still needed to improve the TRAP staining in the bone sections.

In conclusion of our findings, we propose a novel anti-osteoporotic mechanism of FLL in OVX rats (Figure 6C). Ovariectomy triggers Nox4 overexpression followed by ROS overproduction and NF-κB activation as well as mitochondria dysfunction, which initiates and accelerates osteoporosis via the Nox4/ROS/NF-κB pathway. Administration of FLL to OVX rats improves bone quality through inhibiting NF-κB activation and then scavengering abundant ROS via preventing Nox4 expression as well as resuming mitochondria function, suggesting FLL may serve as a natural antioxidant agent against osteoporosis by a direct regulation of Nox4/ROS/NF-κB signaling in OVX rats. The present study also indicates that FLL aqueous extract contains many antioxidant substances which warrants further investigation of the anti-resorption contribution of each constitute.

## Ethics statement

This study was carried out in accordance with the recommendations of “Guide to the Care and Use of Experimental Animals, Animal Care Committee of BUCM, China.” The protocol was approved by the “Animal Care Committee of BUCM, China.”

## Author contributions

LW, RM, MF, DB, SG, and DWZ conceived and designed study; LW, RM, YG, JS, HL, RZ, CL, JT, LL, BC, DDZ, FM, GJ, and JL conducted study; LS, MF, JN, and DWZ analyzed data; LW, RM, YG, DB, and DWZ wrote the paper. DWZ had primary responsibilities for final content. All authors read and approved the final manuscript.

## Funding

This work was supported by Grants from Beijing Municipal Natural Science Foundation (7172126), National Natural Science Foundation of China (NSFC81273995) and the 111 project of MOE (B07007). The funding agencies have no roles in the study design; in data collection, analysis and interpretation; in the writing of the report; and in the decision to submit the article for publication.

### Conflict of interest statement

The authors declare that the research was conducted in the absence of any commercial or financial relationships that could be construed as a potential conflict of interest.
