# Corrigendum: Antioxidant Effect of Fructus Ligustri Lucidi Aqueous Extract in Ovariectomized Rats Is Mediated through Nox4-ROS-NF-κB Pathway

**DOI:** 10.3389/fphar.2017.00590

**Published:** 2017-08-25

**Authors:** Lili Wang, Rufeng Ma, Yubo Guo, Jing Sun, Haixia Liu, Ruyuan Zhu, Chenyue Liu, Jun Li, Lin Li, Beibei Chen, Liping Sun, Jinfa Tang, Dandan Zhao, Fangfang Mo, Jianzhao Niu, Guangjian Jiang, Min Fu, Dieter Brömme, Dongwei Zhang, Sihua Gao

**Affiliations:** ^1^Cell and Biochemistry Lab, Preclinical Medicine School, Beijing University of Chinese Medicine Beijing, China; ^2^Chinese Material Medica School, Beijing University of Chinese Medicine Beijing, China; ^3^Modern Research Center for TCM, Beijing University of Chinese Medicine Beijing, China; ^4^The First Affiliated Hospital of He'nan TCM University Zhengzhou, Henan, China; ^5^Diabetes Research Center, Beijing University of Chinese Medicine Beijing, China; ^6^The Research Institute of McGill University Health Center Montreal, QC, Canada; ^7^Oral Biological Medicinal Science, University of British Columbia Vancouver, BC, Canada

**Keywords:** *Fructus Ligustri Lucidi*, ovariectomy, NADPH oxidase 4 (Nox4), nuclear factor kappa B (NF-κB), oxidative stress

In the original article, there was a mistake in Figure 4. The representative images of immunohistochemical staining (**A–D**; sections were counterstained with hematoxylin; original magnification, X20), and western blot assays (**E, F**) showed that FLL treatment decreased Nox4 expression in tibias and uteri of OVX rats (*n* = 9). In addition, FLL treatment also decreased cytochrome C (Cyto-C; **G**) and increased Bcl-2 expression **(H)** in the tibias of OVX rats as published. The images of the western blot in the Figure 4G were carelessly repeated with Figure 4H. The corrected Figure 4G appears below. The authors apologize for this error and state that this does not change the scientific conclusions of the article in any way.

**Figure 4:**
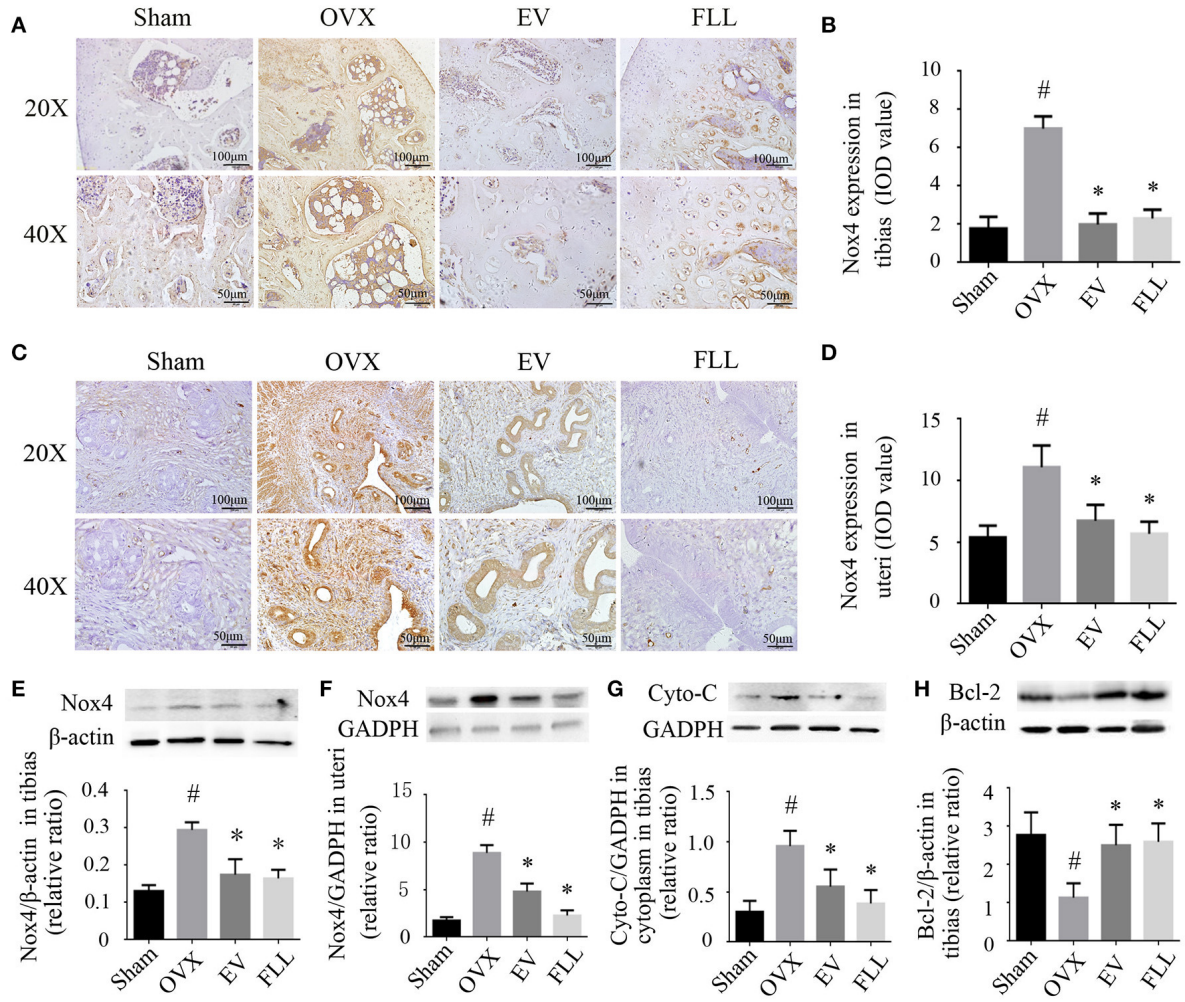


Statistical analysis: The results were expressed as mean ± SD. One-way ANOVA test was performed between multiple groups when homogeneity of variance and normality were met using SPSS software (Version 20.0). Otherwise, *Dunnett*'s T3 and nonparametric tests were conducted between multiple groups, respectively. A value of *p* < 0.05 was considered to be statistical difference.

## Conflict of interest statement

The authors declare that the research was conducted in the absence of any commercial or financial relationships that could be construed as a potential conflict of interest.

